# Leukocyte Telomere Length and Cross‐Sectional and Longitudinal Variations in Gray Matter Volume and Cortical Thickness

**DOI:** 10.1002/brb3.71608

**Published:** 2026-07-14

**Authors:** Ezgi Yetim, Mehmet Akif Topcuoglu, Nuket Yurur Kutlay, Ajlan Tukun, Kader Karli Oguz, Ethem Murat Arsava

**Affiliations:** ^1^ Department of Neurology Faculty of Medicine Hacettepe University Ankara Turkey; ^2^ Department of Medical Genetics School of Medicine Ankara University Ankara Turkey; ^3^ Department of Medical Genetics Duzen Laboratory Group Ankara Turkey; ^4^ Division of Neuroradiology Department of Radiology UC Davis Medical Center Sacramento California USA

**Keywords:** brain aging, cortical thickness, gray matter volume, telomere

## Abstract

**Background:**

Telomere length (TL) has emerged as a candidate predictor for biological aging and age‐associated conditions, yet its relationship with structural brain changes remains incompletely characterized. This study aimed to investigate the cross‐sectional and longitudinal associations between TL, brain gray matter volume (GMV), white matter volume, and cortical thickness (CT) and to examine individual variability in age‐related brain changes.

**Methods:**

The study included 196 cognitively healthy individuals aged ≥50 years. All participants underwent baseline MRI and TL measurement from leukocytes. Among them, 46 individuals had a second MRI scan after an average of 4.7 years. Associations between TL and brain structural measures were assessed using multivariate models adjusting for demographic and imaging confounders. Longitudinal volumetric changes were modeled using region‐of‐interest analyses, whereas regional CT changes were examined using surface‐based methods in FreeSurfer.

**Results:**

The mean age of the cohort was 65.9 ± 8.6 years; 60.9% were women. Median TL was 7.0 kB (interquartile range [IQR]: 6.0–8.4). No significant associations were found between TL and global brain measures in the cross‐sectional analysis. However, longitudinal analysis revealed a significant inverse correlation between TL and GMV decline over time (*r* = −0.370, *p* = 0.012), which remained robust after adjusting for age, sex, follow‐up duration, and baseline GMV (standardized *β* = −0.32). No significant association was found between TL and overall cortical thinning; however, at the regional level, shorter TL was significantly associated with cortical thinning in the left superior parietal lobule.

**Conclusion:**

TL may serve as a predictor of age‐dependent structural brain changes, with the left superior parietal cortex appearing particularly susceptible within this telomere‐brain aging interaction.

## Graphical abstract

In 196 healthy older adults, shorter leukocyte telomere length predicted greater 5‐year global gray matter volume change and regional cortical thinning in the left superior parietal lobule, independent of age and sex.

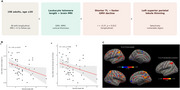

## 1 Introduction

Situated at chromosomal termini, telomeres are repetitive nucleotide sequences that safeguard chromosomal ends against degradation and prevent aberrant end‐to‐end chromosomal fusions. With each cell division, telomere length (TL) undergoes progressive erosion; once it falls below a critical threshold, its protective capacity is compromised, ultimately triggering cellular senescence. Accordingly, progressive TL shortening has been regarded as a hallmark of the organism's biological aging trajectory (Blackburn et al. 2015; Harley et al. 1990).

The association between aging and loss of brain volume is a well‐known entity modulated by individual lifestyle characteristics and vascular risk factors, as well as environmental and genetic factors. Brain resistance represents the brain's ability to withstand senescent brain changes within the normal aging process or neuropathological changes during the pathological aging process (Bocancea et al. 2021; Montine et al. 2019). Brain resilience refers to preserving the brain's integrity despite age‐dependent molecular changes or neuropathology (Arenaza‐Urquijo and Vemuri 2018; Hohman et al. 2016). Resistance and resilience are modulated by some risk/protective factors, including sex, vascular risk factors, education, physical activity, lifestyle enrichment, and genetics (Bocancea et al. 2021). In this perspective, leukocyte TL is closely related with many of these resistance/resilience‐associated factors, with age being the most significant, along with gender (Gardner et al. 2014), nutrition (Paul 2011), physical activity (Ludlow et al. 2008), obesity, smoking (Valdes et al. 2005), depression (Ridout et al. 2016), diseases, and environmental exposures (Kahl et al. 2015). On the other hand, TL also contributes to the genetic underpinnings of brain resilience. Consequently, this multidimensional interplay impacts biological aging and contributes to variations among individuals in terms of age‐related changes in brain structure.

Structural neuroimaging studies have consistently documented a substantial age‐associated decline in cerebral gray matter volume (GMV) (Good et al. 2001; Jernigan et al. 2001; Raz et al. 2005; Resnick et al. 2003; Smith et al. 2007). Research indicates that certain regions exhibit greater susceptibility to age‐related changes in GMV, with significant volumetric reductions in the caudate, hippocampus, cerebellum, inferior frontal, and inferior parietal cortices (Raz et al. 2005; Resnick et al. 2003). Considering structural brain changes from the biological age point of view, TL has been evaluated as a significant determiner for global brain volume decline (Gampawar et al. 2022; Topiwala et al. 2023). In addition, certain regional variations have been highlighted in the relationship between TL and brain atrophy in cross‐sectional studies (King et al. 2014). Yet longitudinal evidence on the influence of TL upon global and regional structural brain features remains limited and inconclusive. The present study therefore sought to characterize the role of baseline TL as a candidate brain resilience parameter in determining structural brain changes among cognitively intact adults, both cross‐sectionally and across a 5‐year prospective interval.

## 2 Methods

### 2.1 Study Participants

The recruitment process and eligibility criteria of the cohort have been detailed previously (Yetim et al. 2021). Briefly, 196 noninstitutionalized adults aged 50 years or above with no prior stroke or established neurological disorder were recruited via print and digital media. Comprehensive medical histories were obtained, and individuals diagnosed with malignancy, chronic hepatic, or renal disease were excluded. Demographic characteristics and vascular risk factor profiles were systematically recorded. All eligible participants underwent MRI examination, and those found to have incidental territorial infarcts—despite no clinical stroke history—were excluded from the study. Forty‐six of the individuals without any major health events during the follow‐up period were evaluated with a second structural brain MRI after an average of 4.7 years.

### 2.2 Measurement of TL

The specifics of TL analyses were thoroughly discussed in our prior publications (Yetim et al. 2021). At the baseline visit, 20 mL of peripheral venous blood was obtained per participant in two EDTA‐anticoagulated tubes for leukocyte TL assessment. Genomic DNA was isolated using the MasterPure DNA Purification Kit (Epicenter) and quality‐verified by 1% agarose gel electrophoresis. At least 3 µg of DNA underwent restriction digestion with HinfI and HpHI endonucleases. Terminal restriction fragments (TRF) were separated by electrophoresis on a 0.5% agarose gel at 2–2.25 V/cm overnight. The gel was sequentially treated by depurination, denaturation, and neutralization prior to membrane transfer via the Whatman TurboBlotter Rapid Downward Transfer System. Hybridization probes were then prepared: The telomere‐specific probe was 3′‐end‐labeled with digoxigenin (DIG), whereas 1 kb and λ‐HindIII ladder probes were DIG‐labeled via random‐prime synthesis. The cross‐linked membrane was incubated in hybridization solution at 37°C for approximately 72 h on a rotating hybridization oven. TRF lengths were subsequently quantified on transparency film by chemiluminescence detection against ladder references (Kimura et al. 2010). All analyses of TRF length were carried out blinded the complete clinical data.

### 2.3 Imaging Data Acquisition and Analysis

Structural MRI data were acquired using a 1.5‐T scanner (Magnetom Symphony TIM, Siemens, Erlangen, Germany). High‐resolution three‐dimensional (3D) T1‐weighted images were acquired using a magnetization‐prepared rapid gradient‐echo (MPRAGE) sequence with 1 mm isotropic voxel size (slice thickness = 1 mm). All volumetric analyses were performed on these 3D T1‐weighted MPRAGE images. Importantly, both cross‐sectional and longitudinal MRI data were acquired using the same scanner and identical imaging parameters to ensure consistency across time points. Sequences were automatically processed using FreeSurfer (version 7.4.1) recon‐all procedure to reconstruct cortical surfaces and to get segmented region‐of‐interest volumes. Initial preprocessing included intensity normalization, skull stripping, and tissue segmentation (Dale et al. 1999; Fischl et al. 1999). Surface‐based smoothing was applied with a full‐width/half‐maximum of 15 mm. GMV and cortical thickness (CT) were extracted for each subject following the reconstruction of the cortical surface using FreeSurfer's automated pipeline (Greve et al. 2014). Total white matter volume was derived from FreeSurfer's automated segmentation and included labeled white matter hypointensities as part of the estimate, consistent with standard FreeSurfer output conventions. Finally, the vertex‐wise mean of each cortical Desikan–Killiany ROI was calculated for regionally specified analyses (Desikan et al. 2006). For group‐level statistical analysis, FreeSurfer's glmfit feature that uses general linear model (GLM) was employed. Longitudinal surface‐based analysis was used to determine the relationship between TL and cortical thinning regionally (Wikgren et al. 2014). Age and sex were included in the model to control for potential confounding effects. Statistical significance was assessed using cluster‐based correction for multiple comparisons with a Monte Carlo simulation, ensuring robust inferences.

### 2.4 Statistical Analysis

Categorical variables are expressed as *n* (%) and continuous variables as mean ± standard deviation (SD) or median (interquartile range [IQR]). Group‐wise comparisons were performed by chi‐square test for categorical variables and Student's *t*‐test or Whitney *U* test for continuous variables depending on the normality of the distribution. Associations between structural gray matter change parameters and TL were assessed using Pearson correlation. The Wilcoxon signed‐rank test was used to compare the observations between two visits in the cohort. Finally, linear regression models were constructed to determine independent factors related to the rate of GMV decrease. Age, sex, TL, and basal GMV were introduced into the models as independent variables. A *p* value of <0.05 was considered statistically significant. Statistical analyses were performed by using R version 4.4.2 (R Core Team 2024).

## 3 Results

A total of 196 participants were included in the study, with 46 individuals having follow‐up imaging. The mean ± SD age of the entire cohort was 65.9 ± 8.6 years. Participants with follow‐up visits were significantly younger than those without follow‐up (62.3 ± 6.7 vs. 67.2 ± 8.8 years, *p* < 0.001). The proportion of females was comparable between groups (60.9% vs. 60.8%). The median (IQR) TL was similar across groups, with an overall value of 7.0 (6.0–8.4) kB for the entire cohort. The rates of hypertension, diabetes mellitus, coronary artery disease, hyperlipidemia, and smoking did not differ substantially between groups (Table 1). The groups showed no significant differences in terms of neuroimaging metrics, including GMV, white matter volume, total subcortical GMV, and mean CT (Table 1).

**TABLE 1:** Baseline characteristics of the study population stratified according to follow‐up status.

	All (*n* = 196)	No follow‐up (*n* = 150)	With follow‐up (*n* = 46)
Age (years)	65.9 ± 8.6	67.2 ± 8.8***	62.3 ± 6.7***
Female (%)	60.9	60.9	60.8
Telomere length (kB)	7.0 (6.0–8.4)	7.0 (6.0–8.2)	7.0 (6.5–9.0)
Hypertension (%)	54.6	55.5	52.9
Diabetes (%)	20.3	20.0	21.6
Hyperlipidemia (%)	50.2	47.7	58.8
Coronary artery disease (%)	22.2	23.2	19.6
Smoking (%)	24.6	26.5	19.6
Total GMV (cm^3^)	423.5 ± 44.5	420.7 ± 46.0	431.7 ± 39.0
Subcortical GMV (cm^3^)	51.0 ± 4.7	50.8 ± 4.8	51.7 ± 4.2
White matter volume (cm^3^)	455.7 ± 5.5	453.4 ± 57.2	462.3 ± 49.0
Mean cortical thickness (mm)	2.40 ± 0.13	2.39 ± 0.14	2.43 ± 0.09

Abbreviation: GMV, gray matter volume.

***p < 0.001.

In cross‐sectional analyses, no significant correlation was found between TL and GMV or CT. Participants with the lowest quartile of TL had significantly lower CT (median [lowest quartile] = 2.36 [2.31 − 2.45] and median [other quartiles] = 2.43 [2.34 − 2.50]; *p* = 0.046). However, when controlling for age and sex in a linear regression model, the relationship between TL quartile and CT was no longer significant (*β* = −0.02, *p* = 0.18). Furthermore, surface‐based analysis using FreeSurfer's GLM framework revealed no significant regional associations between TL and CT following cluster‐wise correction for multiple comparisons. Volumetric comparisons across TL quartile groups also demonstrated no significant differences in total GMV, cortical volume, or subcortical GMV (all *p* > 0.10).

**FIGURE 1:**
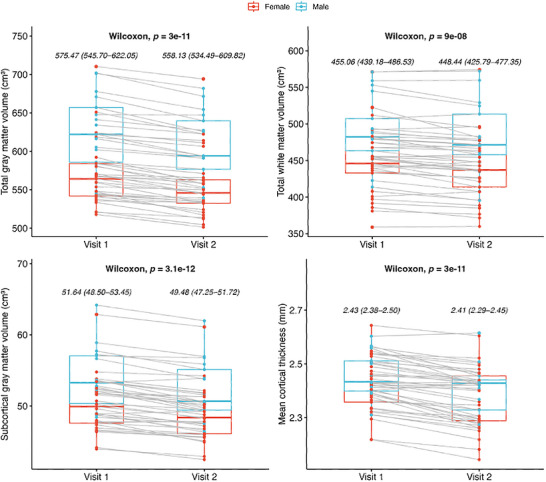
The distribution of total gray matter, white matter, subcortical gray matter volumes, and mean cortical thickness at baseline and follow‐up, color‐coded by sex. The numerical values presented next to box plots represent the overall median and IQR at each time point.

**FIGURE 2:**
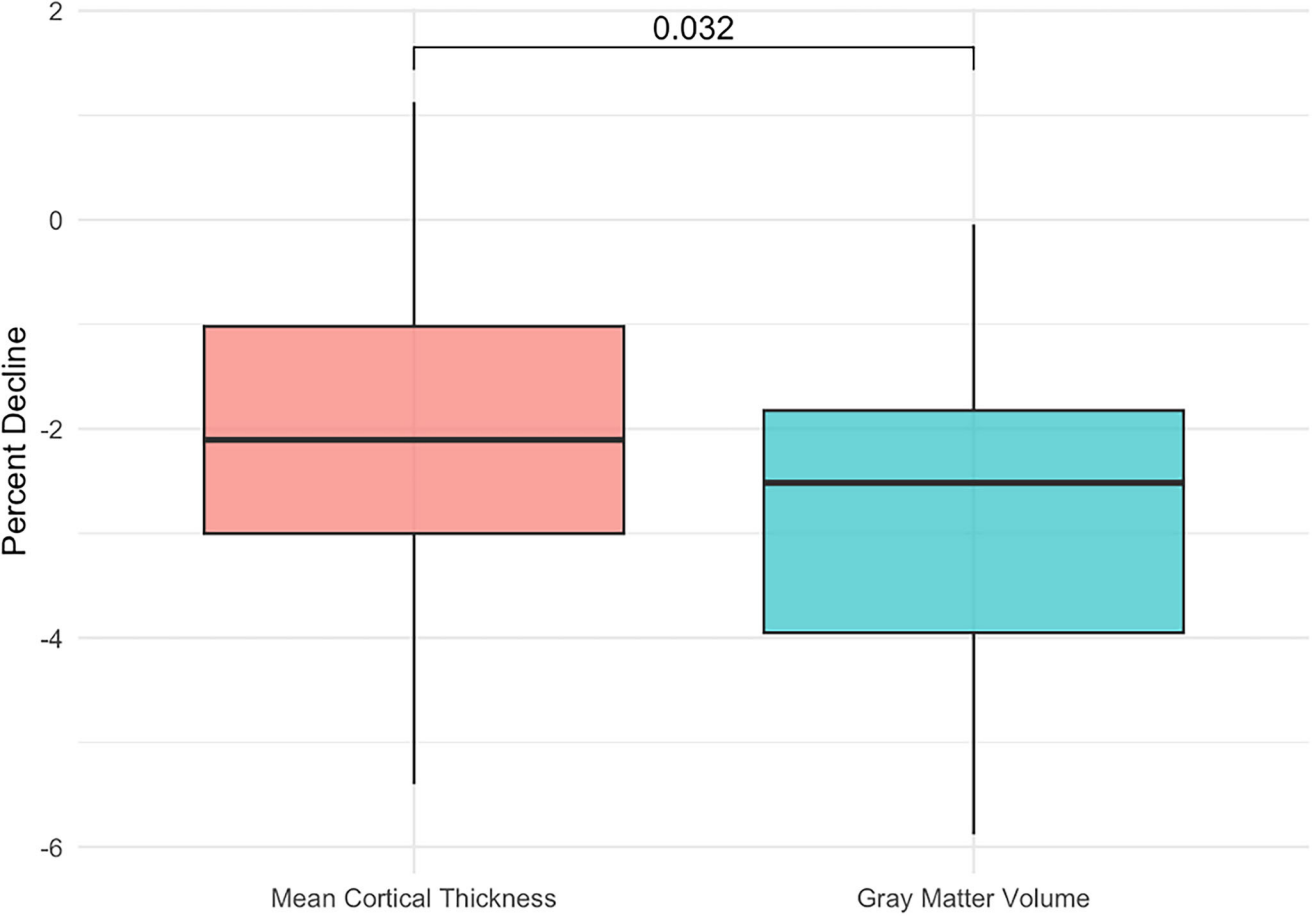
The percent decline in mean CT and GMV during follow‐up.

In the longitudinal data, we observed 2.81% ± 1.47% reduction in GMV, 2.01 ± 2.14 in white matter volume, 2.92 ± 1.82 in subcortical GMV, and 2.12 ± 1.53 in CT over a mean follow‐up of 4.7 ± 0.45 years (Figure 1). All these reductions were statistically significant. The mean percent decline for GMV was significantly larger (more negative) than that for mean CT (*t* (85.86) = 2.18, *p* = 0.032; Welch's *t*‐test) (Figure 2). As GMV is the product of CT and surface area, this additional volumetric decline is likely attributable to concurrent reduction in surface area, a component not independently available for analysis in this dataset.

**FIGURE 3:**
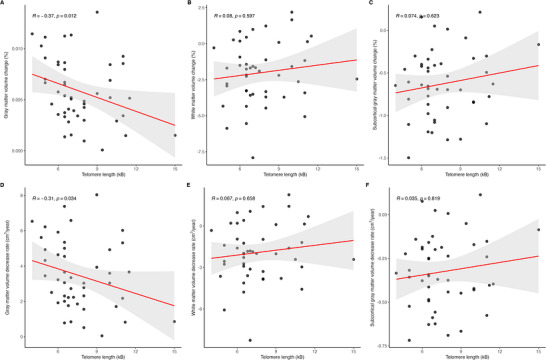
Correlations between TL and longitudinal brain volume changes. Scatter plots showing the relationship between TL (kB) and brain volume change metrics in 46 participants. The top row depicts percent change in gray matter (A), white matter (B), and subcortical gray matter (C) volumes over the follow‐up period. The bottom row depicts annualized volume decrease rates (cm^3^/year) for gray matter (D), white matter (E), and subcortical gray matter (F). Red lines represent linear regression fits with 95% confidence intervals (gray shading). Spearman's correlation coefficients (*R*) and *p* values are displayed in each panel. Significant correlations were observed between TL and gray matter volume change (*R* = −0.37, *p* = 0.012) and gray matter volume decrease rate (*R* = −0.31, *p* = 0.034), indicating that longer telomeres are associated with less gray matter atrophy over time.

TL displayed significant negative correlations with the overall percent decline in GMV (*r* = −0.37, *p* = 0.012) and the annual GMV decrease rate (mm^3^/year) (*r* = −0.31, *p* = 0.034) (Figure 3). No significant association between TL and longitudinal changes in white matter volume or subcortical GMV was evident (Figure 3). In multivariate models adjusted for age, sex, baseline GMV, and interscan interval, percent GMV change and annual GMV decline were both negatively associated with TL (*β* = −0.32, *p* < 0.05 and *β* = −0.31, *p* < 0.05, respectively) (Tables 2 and 3). TL showed no significant association with longitudinal changes in white matter volume (*β* = 0.036, *p* = 0.807) or subcortical GMV (*β* = 0.108, *p* = 0.468) in multivariate models.

**TABLE 2:** Multivariate regression model with gray matter volume (GMV) percent change introduced as the dependent variable model.

	*β* ^1^	Std. error	*t* value	*p* value	*β* ^2^
Intercept	−0.00397	0.00928	−0.428	0.671	
TL	−0.00371	0.00161	−2.298	**0.027**	−0.32
Age	0.00017	0.0000698	2.440	**0.019**	0.34
Female gender	0.00037	0.00105	0.349	0.729	0.06
Baseline total GMV	0.0000000232	0.00000000969	2.391	**0.022**	0.37
Interval between visits	−0.00013	0.000087	−1.487	0.145	−0.22

*Note*: Residual standard error: 0.002825 on 40 degrees of freedom; multiple *R* ^2^: 0.317, adjusted *R* ^2^: 0.231; *F*‐statistic: 3.713 on 5 and 40 DF; *p* value: 0.007453; *β* ^1^: unstandardized regression coefficient; *β* ^2^: standardized regression coefficient. Telomere length was introduced into the model after log normalization.

Abbreviations: TL, telomere length; VIF, variance inflation factor.

Bold values indicate statistical significance (*p* <0.05).

**TABLE 3:** Multivariate regression model with annual gray matter volume (GMV) decline introduced as the dependent variable model.

	*β* ^1^	Std. error	*t* value	*p* value	*β* ^2^
Intercept	−5490.8	5473.0	−1.00	0.322	
TL	−2133.5	952.6	−2.24	**0.031**	−0.31
Age	98.6	41.2	2.39	**0.021**	0.33
Female gender	162.2	618.5	0.26	0.795	0.04
Baseline total GMV	0.02	0.01	3.40	**0.002**	0.52
Interval between visits	−79.7	51.4	−1.55	0.129	−0.22

*Note*: Residual standard error: 1667 on 40 degrees of freedom; multiple *R* ^2^: 0.3545; adjusted *R* ^2^: 0.2738; *F*‐statistic: 4.393 on 5 and 40 DF; *p* value: 0.002787; *β* ^1^: unstandardized regression coefficient; *β* ^2^: standardized regression coefficient. Telomere length was introduced into the model after log normalization.

Abbreviations: TL, telomere length; VIF, variance inflation factor.

**FIGURE 4:**
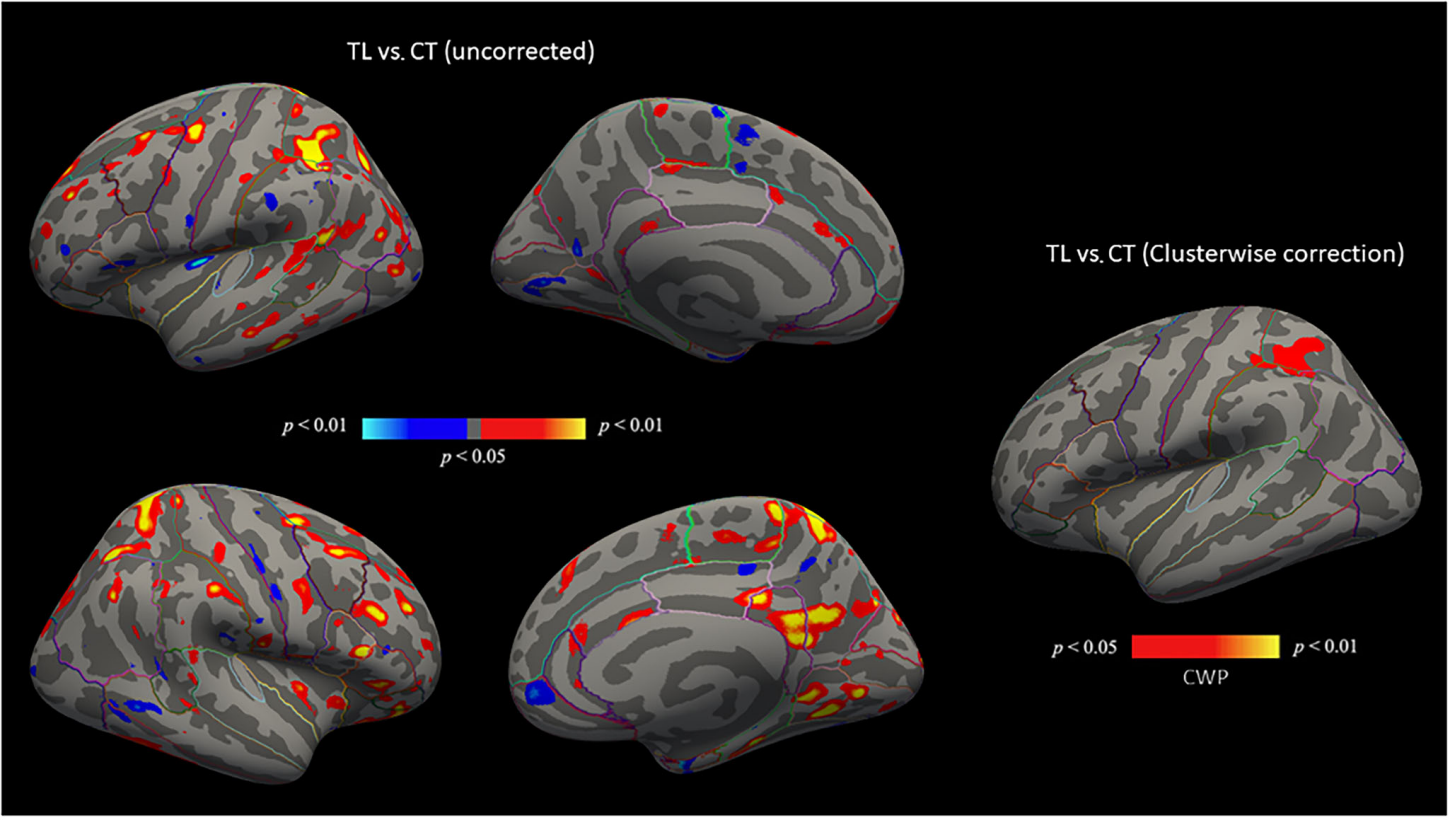
Surface‐based analyses of change in mean cortical thickness and TL. The model is adjusted for age. The association between change in cortical thickness and TL remains significant only for the left superior parietal lobule after cluster‐wise correction. TL, telomere length.

No significant association has been found between CT and TL in either the cross‐sectional analyses or the longitudinal models; however, longitudinal surface‐based analysis revealed significant relationships between CT and TL throughout various regions of the brain. Following cluster‐wise correlation, significant associations persisted only in the left superior parietal lobule (SPL) (Figure 4).

## 4 Discussion

The present study reveals an intricate interplay between TL and age‐associated structural brain alterations. Cross‐sectional analyses yielded no significant associations, whereas longitudinal follow‐up demonstrated that TL was meaningfully related to both overall GMV loss and cortical thinning in the left SPL. Collectively, these findings suggest TL as a candidate biomarker for individual brain resilience and age‐related structural brain change. The regional association with CT, however, warrants cautious interpretation, given its exploratory nature and the absence of cross‐regional effect size comparisons.

Previous research on the cross‐sectional relationship between TL and structural brain features, such as GMV, CT, and white matter hyperintensities, has yielded mixed results with some reporting associations with global or subcortical volumes (King et al. 2014; Topiwala et al. 2023; Wikgren et al. 2014), and others found no significant relationship (Cao et al. 2023). A meta‐analysis revealed only a weak positive correlation between TL and hippocampal volume (Nilsonne et al. 2015). Another recent systematic review and meta‐analysis integrating data from 27 studies confirmed that longer leukocyte TL is significantly associated with higher total brain and hippocampal volumes as well as better cognitive performance, although no association was found with white‐matter hyperintensities (Gampawar et al. 2022). In our study, TL showed no significant cross‐sectional association with structural brain measures, likely reflecting the biological complexity of aging and the influence of genetic and environmental factors that might affect the brain structure and therefore obscure the direct relationship between TL and GMV or CT (Pathak et al. 2021).

Unlike cross‐sectional approaches, longitudinal subject evaluation can attenuate the inter‐individual variability noted above and may thus provide more insights into TL's role as a marker of biological aging. Limited evidence in this area has suggested an increased loss in hippocampal volume among subjects with greater telomere attrition (Staffaroni et al. 2018), despite the lack of an association between TL and memory system structures in cross‐sectional analyses. The current results broaden this observation to encompass total gray matter architecture. From a biological perspective, telomere shortening is thought to contribute to brain aging through converging pathways, including cellular senescence, mitochondrial dysfunction, and neuroinflammation (Sahin et al. 2011; Schank et al. 2020; Zhu et al. 2019). Although the precise mechanisms linking peripheral leukocyte TL to structural brain changes remain to be fully elucidated, the present findings support TL as a relevant systemic marker of biological aging with implications for brain integrity and warrants further investigation.

Notably, in cross‐sectional analyses, no meaningful relationship was identified between TL and overall GMV change, yet no discrete anatomical region demonstrated a significant association with TL in surface‐based analyses. In longitudinal analyses, the magnitude of cortical thinning in the left SPL was significantly associated with TL, despite an absence of relationship between TL and the tempo of changes in global CT. This divergence between GMV and CT findings likely reflects inherent differences in how each metric captures age‐related atrophic processes. Volume is determined by incorporating both the surface area and thickness parameters, meaning that alterations in either parameter may yield a more significant result than changes in thickness alone. Consequently, GMV may better capture the full extent of age‐related atrophy. A longitudinal study by Sele et al. (2021) demonstrated greater inter‐individual variability in GMV decline compared to CT, supporting this notion. Furthermore, CT demonstrates greater sensitivity to age‐related changes observed in specific regions, notably the parietal cortex, whereas the decline in GMV is more widespread (Lemaitre et al. 2012). These observations are consistent with our data, in which TL—as a biological aging indicator—reflected chronological aging by associating with both global volumetric and regional CT measures. Of note, the participants in this cohort were cognitively unimpaired individuals in late midlife or early older age, a demographic in which cortical thinning proceeds relatively slowly. Published data indicate that in healthy individuals aged 44–49 years, CT declines at a mean annual rate of 0.26% on the left and 0.17% on the right hemisphere (Shaw et al. 2016). However, GMV reductions become more pronounced in older populations and are more strongly linked to cognitive decline than cortical thinning (Fleischman et al. 2014). Prior research by Resnick et al. (2003) found that GMV declines at an annual rate of 1.7% in the frontal lobe, 1.4% in the parietal lobe, 0.8% in the temporal lobe, and 0.9% in the occipital lobe. The comparatively modest magnitude of CT change may, therefore, account for the absence of significant global CT findings over the 5‐year observation window in this cohort.

Our longitudinal finding that cortical thinning in the left SPL was significantly associated with TL aligns with recent evidence that highlights this region's heightened vulnerability to age‐related structural changes. Longitudinal studies have consistently demonstrated that the superior parietal cortex is among the regions with the highest rates of cortical thinning during normal aging, exhibiting a pronounced anterior–posterior gradient where frontal and parietal areas decline more than temporal and occipital regions (Thambisetty et al. 2010). Furthermore, recent functional connectomics studies indicate that fronto‐temporo‐parietal hubs, including the SPL, are central nodes within high‐demand cognitive networks, characterized by dense interconnectivity and high metabolic burden. These features may predispose them to cumulative cellular stress and age‐related attrition (Filippi et al. 2023; Xu et al. 2022). In this light, our results suggest that TL may act as a peripheral biomarker reflecting the selective vulnerability of cortices with high connectivity demands.

This study has several limitations that merit acknowledgment. To begin with, the overall sample was limited in size, especially in the longitudinal subgroup, potentially constraining statistical power to detect more nuanced associations. Additionally, a follow‐up interval of approximately 5 years may have been insufficient to capture the full spectrum of age‐related brain changes, particularly for CT. Furthermore, TL was assessed only at baseline, precluding any direct quantification of telomere attrition across the study period. Lastly, despite adjustment for principal confounders, including age, sex, and baseline GMV, residual confounding from lifestyle factors, genetic background, and environmental exposures cannot be excluded.

## 5 Conclusion

The present findings indicate that TL may constitute a valuable biomarker of brain senescence, with practical relevance for stratifying individuals according to neurodegenerative risk. The region‐specific pattern of TL‐related associations underscores the complexity of telomere biology in the central nervous system and supports the need for future longitudinal investigation to clarify the underlying mechanistic pathways.

## Author Contributions

**Ezgi Yetim** was responsible for conceptualization, methodology, software, data curation, investigation, formal analysis, visualization, and writing the original draft. **Mehmet Akif Topcuoglu** contributed to methodology, data curation, supervision, and writing, review, and editing. **Nuket Yurur Kutlay** contributed to methodology, formal analysis, and writing, review and editing. **Ajlan Tukun** contributed to methodology, formal analysis, and writing, review and editing. **Kader Karli Oguz** contributed to methodology, validation, supervision, and writing, review and editing. **Ethem Murat Arsava** contributed to conceptualization, methodology, supervision, funding acquisition, and project administration and was involved in writing, review, and editing.

## Funding

The study was funded by the Turkish Academy of Sciences Young Scientists Award Program (GEBIP) awarded to EMA.

## Ethics Statement

Approval was obtained from the ethics committee of Hacettepe University (REC number: GO 13/243). The procedures used in this study adhere to the tenets of the Declaration of Helsinki.

## Consent

Written informed consent was obtained from study participants.

## Conflicts of Interest

The authors declare no conflicts of interest.

## Data Availability

Data from this study will be made available upon reasonable request to the corresponding author.
